# Evolving child and adolescent mental health and development programs in Chile

**DOI:** 10.26633/RPSP.2019.33

**Published:** 2019-04-17

**Authors:** Flora de la Barra, Matias Irarrazaval, Ana Valdes, Gonzalo Soto-Brandt

**Affiliations:** 1 University of Chile University of Chile Faculty of Medicine Eastern Mental Health Department Santiago Chile Eastern Mental Health Department, Faculty of Medicine, University of Chile, Santiago, Chile.; 2 Millennium Institute for Research in Depression and Personality Millennium Institute for Research in Depression and Personality Santiago Chile Millennium Institute for Research in Depression and Personality, Santiago, Chile.; 3 South East Metropolitan Health Service South East Metropolitan Health Service Mental Health Network Santiago Chile Mental Health Network, South East Metropolitan Health Service, Santiago, Chile.; 4 Ministry of Health Ministry of Health Mental Health in Primary Care Division Santiago Chile Mental Health in Primary Care Division, Ministry of Health, Santiago, Chile.

**Keywords:** Mental health services, child health services, adolescent health services, national health programs, Chile, Servicios de salud mental, servicios de salud del niño, servicios de salud del adolescente, programas nacionales de salud, Chile, Serviços de saúde mental, serviços de saúde da criança, serviços de saúde do adolescente, programas nacionais de saúde, Chile

## Abstract

This analysis reviews the situation of child and adolescent mental health in Chile, organizational determinants, and the initiatives and interventions implemented to enhance child development despite the country’s inequities. Progressive development of national mental health plans is covered, from the country’s first plan in 2000, to growing the number of mental health professionals and the training they receive, such as MhGAP, to the implementation of “Chile Crece Contigo,” whose preliminary evaluations are starting to show some effectiveness. However, the World Health Organization reports that progress in complying with the United Nations Convention of Children’s Rights is insufficient. A set off legislative initiatives on behalf of children and adolescents have been passed, while others are being discussed in Parliament. There is much to be done in the nation as a whole and within its health system to ensure improved child and adolescent mental health and wellbeing. More research into child and adolescent mental health should be undertaken. Adequate funding and policymaking are also crucial to giving priority to child and adolescent mental health in Chile.

## RESUMEN

En este análisis se examina la situación de la salud mental de niños y adolescentes en Chile, los determinantes institucionales y las iniciativas e intervenciones ejecutadas para mejorar el desarrollo infantil a pesar de las inequidades del país. Se ha abarcado el desarrollo progresivo de los planes nacionales de salud mental, desde el primer plan nacional en el año 2000, pasando por el aumento del número de profesionales de salud mental y la capacitación que reciben, como el programa MhGAP, hasta la ejecución de “Chile Crece Contigo”, cuyas evaluaciones preliminares están comenzando a mostrar cierta eficacia. Sin embargo, la Organización Mundial de la Salud informa que el progreso en el cumplimiento de la Convención de las Naciones Unidas sobre los Derechos del Niño es insuficiente. Se ha aprobado un conjunto de iniciativas legislativas a favor de los niños y adolescentes, mientras que otras están tratándose en el parlamento. Es mucho lo que hay por hacer en el país en general y en su sistema de salud para garantizar el mejoramiento de la salud mental y el bienestar de niños y adolescentes. Es necesario aumentar la investigación sobre la salud mental de niños y adolescentes. La disponibilidad de fondos suficientes y la formulación de políticas son también cruciales para dar prioridad a la salud mental de niños y adolescentes en Chile.

## RESUMO

Uma análise foi realizada para examinar a situação da saúde mental de crianças e adolescentes no Chile, determinantes organizacionais e iniciativas e intervenções implementadas para melhorar o desenvolvimento infantil diante das iniquidades no país. É descrita a evolução dos planos nacionais de saúde mental, a partir do primeiro plano elaborado em 2000, o crescimento do número de profissionais de saúde mental e a capacitação recebida, como parte do programa mundial de ação para reduzir as lacunas em saúde mental da OMS (MhGAP), e a implementação do programa *Chile Crece Contigo* cujas avaliações preliminares indicam certo grau de efetividade. Porém, segundo informou a Organização Mundial da Saúde (OMS), houve pouco progresso no cumprimento da Convenção das Nações Unidas sobre os Direitos da Criança. Um conjunto de iniciativas legislativas em prol das crianças e adolescentes foi aprovado e outras propostas estão em debate no congresso. Há ainda muito a ser feito no país como um todo e dentro do sistema de saúde para garantir melhor saúde mental e bem-estar para crianças e adolescentes. Devem ser realizadas outras pesquisas sobre saúde mental da criança e do adolescente. É indispensável dispor de financiamento e políticas adequados que priorizem a saúde mental da criança e do adolescente no Chile.

With recent efforts to meet the United Nations’ Sustainable Developmental Goals (SDGs), the World Health Organization (WHO) has been promoting mental health and well-being for all at all ages, specifically drawing more attention to the mental health needs of young people (1). Children and adolescents comprise nearly one-half of the world’s population, yet receive only a small fraction of health resources. Today, there are evidence-based preventive interventions that can be delivered throughout the life-course to help prevent mental disorders in children and adolescents (2).

The total estimated population of Chile is just over 17.5 million inhabitants, 27.1% of whom are under 19 years of age (3). Although Chile has recently been classified as a high-income country, persistent inequities are a concern. Poverty, which has a dramatic impact on behaviors, emotions, and mental health, is higher among the child population of Chile (22.4%) than it is among its other vulnerable groups (4); likewise, the burden of disease, measured in disability-adjusted life years (DALYs), is 30.3% for 1 – 9 year olds and 38.3% for 10 – 19 year olds—notably higher than among adults (4).

## CHILD AND ADOLESCENT MENTAL HEALTH

Chile’s health network is divided into 29 territories. Inpatient facilities for critical child and adolescent psychiatry are present in only 15, almost 40% of which are located in the Santiago metropolitan area. Those without psychiatric care are mostly in outlying areas of the country. Ambulatory child and adolescent psychiatric care is divided into 345 smaller territories/counties overseen by local authorities. Of these, only 97 have child and adolescent psychiatric teams, representing 28% coverage (4). In the underserved areas, as well as in rural and first-nation communities, access is limited—children must travel to other counties to receive mental health services (4). According to the Ministry of Health, only 2.4% of Chile’s health care budget is allocated to mental health, a figure similar to that of lower-income countries.

The prevalence of developmental delays among the population under 6 years of age was 25.1% in 2006, higher among children of low socioeconomic status. In 2016, after continuous implementation of early stimulation programs during routine health controls in all of the country’s geographic areas, delays were found in only 11.3% of this population (5).

The prevalence of people being treated for psychiatric disorders in Chile is 6.4%, of which only 1.5% are assisted in specialized centers, while the remaining 4.9% are treated by primary care services. The privately-insured population receives 8 times more ambulatory treatment and 1.5 times more inpatient care than the population with public insurance (4). The 2008 *National Report on Human Resources Gaps* showed that there were 9.2 child and adult psychiatrists per 100 000 population. There are 1.4 child and adolescent psychiatrists per 100 000 inhabitants in the public sector versus 3.4 in the private sector, with unequal distribution throughout the country’s various geographic areas (6).

The largest community study on children and adolescents from 4 – 18 years of age in Chile was done in four different provinces. The Spanish version of the Diagnostic Interview Schedule for Children Version IV interviews yielded a prevalence of 22.6% for mental health disorders with impairment, only one-third of which had been in contact with any type of service (7, 8). The prevalence of alcohol and tobacco use in adolescents from 12 – 18 years of age was 35.5% and 14.1%, respectively, in 2002, and dropped to 18.7% and 5.5% by 2016 (9). UNICEF stated that 15% of adolescents from 13 – 15 years of age in Chile reported having suffered violence in school and 29% had been involved in a physical fight in 2017 (10).

## A NATIONAL MENTAL HEALTH PLAN: THE FIRST STEP

Chile’s first national mental health plan (*Plan Nacional de Salud Mental y Psiquiatría*) for all ages was formulated and implemented in the year 2000. Ascribing to WHO guidelines, the plan promoted the development of a network of mental health services, including some specific actions for children and adolescents. Some key results of the plan’s implementation were providing mental health assistance within the primary health care network, integrating psychosocial teams into general health teams, and establishing specialized facilities in the community. These strategies have sought to increase the availability of mental health care to those in need. Though implemented gradually, most facilities have adopted a community model of assistance. Inpatient care units for adults and adolescents in general hospitals have doubled since 2000, while beds in psychiatric hospitals continue to close.

In 2017, a new expanded plan was implemented. It defined goals to be achieved by 2025 in the following fields of work: regulation and human rights; delivery of mental health services; financing; quality management; information and research; human resources and training; and participation and intersectoral action (11).

Drug prevention programs in schools, implemented jointly by the National Service for Drug & Alcohol Prevention and Rehabilitation and local health teams reach 8.9% of students in all grades. Actions include leaflets for students, guidelines and didactic support material for teachers, and workshops for parents (12).

For the 7 372 public and private subsidized schools in Chile, the Department of School Health (*Junta Nacional de Auxilio Escolar y Becas*) defines a vulnerability index for each student, which determines the psychosocial priority for intervention at each school. A “Skills for Life” program has been implemented at 2 306 schools (31%) considered to be the most vulnerable (13). This intervention includes workshops for students, problem detection, and referrals to the health network (13). Evaluations have shown that the intervention positively impacts school achievement and student behavior (14). In addition, the Ministry of Education of Chile has developed the “Policy for Positive School Relationships 2015 – 2018,” which installed a specialized team in each school to coordinate different programs from other sectors.

In addition, four mental health diagnoses have been included in the National Health Program for Guaranteed Access, Quality, and Funding of Treatment: drug use/abuse for persons under 20 years of age, first episode of schizophrenia for any age, and depression and bipolar disorder for those over 15 years of age (15).

## PRIMARY CARE: ENTRY TO THE MENTAL HEALTH CARE SYSTEM

In recent years, several strategies and prevention programs have been implemented in Chile to strengthen child and adolescent assistance by primary care services within the integral model of family community care. Primary care mental health programs currently reach 20% of 5 – 9 year olds and 29% of 10 – 19 year olds estimated to have disabling psychiatric disorders. The main diagnoses detected, treated, and/or referred by primary care are depression, attention deficit disorder, and mental health problems, such as suicide, maltreatment, and abuse (11).

From 2012 – 2014, there was a 50% increase of mental health human resources, reaching 41.5 per 100 000 public health beneficiaries. At least one psychologist and a social worker have been integrated into each primary care health team serving a designated segment of the population. These staff carry out mental health promotion and preventive strategies during health check-ups, parental skills workshops at the health centers, and home and school visits. During these activities, they also perform detection, psychosocial treatment, and provide referrals for some mental health issues (11).

Professionals assisting children and adolescents with mental health issues are trained in diagnosis, detection, and treatment of mental and substance abuse disorders in non-specialized health settings. In 2016 – 2018, a total of 2 983 of the 66 753 primary care professionals were trained in the WHO Mental Health Gap Action Program (mhGAP; 16). Distance training in mental health topics has also been delivered to 3 680 primary care staff. Furthermore, local teams run training programs that address the specific needs of their area.

Another strategy to train primary care teams to address complex mental health issues has been to conduct periodic consultations with a psychiatrist; this activity was practiced in 80% of primary care centers in 2017. These monthly face-to-face meetings between a specialist and the primary care team were held to discuss complex cases and make decisions on treatment. They provided a continuous training space within clinical practice (17). A study of their impact in 2009 reported that in counties where > 50% of primary care centers were participating, there was a significant reduction of psychiatric hospitalization in the population over 15 years of age (18).

Follow up care programs include continuity of care through home visits, telephone consultations, and coordination of meetings and efforts with schools and other community organizations (11). Preventive mental health activities are also currently being introduced; these are designed specifically for adolescents to take place in a friendly setting within a primary care center (11). In 2016, a mental health care assistance program was implemented for abused/neglected children in protective custody. This program included restructuring and increasing cooperation and communication among the various government sectors involved (11).

The most important and comprehensive preventive child and adolescent mental health program in Chile was created in 2006. This intersectoral system for child development called “Chile Crece Contigo” was the first of its kind in Latin America. It involves several public sectors (health, education, social protection, and justice) in all of Chile’s counties, including rural areas. The initiative delivers coordinated services at the local level from prenatal care (20 weeks) to 4 years of age. In addition, various forms of support are provided for children and families of the poorest 40% and underserved populations. The program is based in the health sector, operating through the Biopsychosocial Development Support Program. Starting with the first prenatal care visit, a set of interventions and social services are offered. Benefits are complementary to other health services and are organized into five dimensions: prenatal care, birth and postpartum, inpatient child, child health control, and remedial interventions for children with development delays (19). “Chile Crece Contigo” has been shown to effectively improve child psychomotor and social skills, reduce inequities, and promote children’s rights (5). Recently, the program added an extension focused on the mental health of children 5 – 9 years of age.

## LEGISLATIVE ACTIONS

Health sector initiatives in Chile are aligned with a growing effort to produce a new National Policy on Children and Adolescents and a set of bills that are progressively building a system aligned with the UN Convention on the Rights of the Child (20). A new Undersecretary of Children within the Ministry of Social Development and an Office of the Ombudsman have endowed the establishment of an Inter-Ministerial Committee to coordinate policies at the highest level. Presently, there is an active legislative agenda with numerous legislative projects to improve child protection. Some have been approved, including the Law of Videotaped Child Interviews Before Courts and the Gender Identity Bill. Others are being discussed, such as the Mental Health Law, a modification of the Adoption Bill, and a new law for children in protective custody or in the juvenile corrective system (21).

## DISCUSSION AND CONCLUSIONS

Chile is making progress in providing and organizing mental health services for children and adolescents; however, funding, especially for human resources, remains insufficient, as does intersectoral coordination. Evaluations of efficacy and research in child and adolescent mental health are still in preliminary stages.

Chile’s commitment to UN Convention on the Rights of the Child has produced some advances, but an acceptable level of protection has not been reached, and further implementation is needed. Progress in mental health care should run parallel to reducing all types of inequities within the country’s whole society, where risk factors for child development originate. Considerable additional work is needed to improve and ensure continuity of the aforementioned mental health programs for children and adolescents. Furthermore, an adequately funded, more comprehensive plan that expands mental health care for at-risk children and prevention for all should be developed.

## Author contributions.

All authors conceived the original idea, collected/analyzed/contributed the data or analysis tools, interpreted the results, and wrote the paper. All authors reviewed and approved the final version.
